# Diet and Physical Activity Behaviors of Families Receiving Maternal and Child Health Services: The Perspective of the Home Visitor

**DOI:** 10.51250/jheal.v2i1.33

**Published:** 2022-03-11

**Authors:** Sydney Miller, Celina H. Shirazipour, Aimee Fata Holmes, Sarah-Jeanne Salvy, Kayla de la Haye

**Affiliations:** 1 Department of Population and Public Health Sciences, University of Southern California, U.S.A.; 2 Research Center for Health Equity, Samuel Oschin Comprehensive Cancer Institute, Cedars-Sinai Medical Center, Los Angeles, CA, U.S.A.; 3 Department of Medicine, University of California Los Angeles, U.S.A.

**Keywords:** physical activity, diet, home visitation services

## Abstract

Women and children enrolled in federally funded home visitation services are at an increased risk for unhealthy diet and physical activity patterns. Home visitors have a privileged relationship with their clients and hold a unique perspective of the multilevel influences surrounding these behaviors. This study explored the question: “What are home visitors’ perspectives and experiences with their families’ diet and physical activity behaviors?” Home visitors enrolled in a larger trial were invited to participate in focus group sessions (n=13). Topics covered their clients’ constraints and capacity building opportunities for healthful diet and activity practices. Reflexive thematic analysis was used to analyze the data. The home visitors discussed key influences on diet and activity, and three overarching themes were identified: (a) acute periods of crises and mental health issues; (b) the role of the mother within the family; and (c) support and barriers within mothers’ broader social network. The themes identified in this study highlight the complex, multidimensional influences on the dietary and physical activity patterns of these families, and pinpoint key areas of opportunity for intervention.

Unhealthy dietary patterns and inadequate levels of physical activity are associated with a myriad of chronic non-communicable diseases (NCD), such as obesity and cardiovascular disease (Centers for Disease Controls and Prevention [CDC], 2019, 2020 a,b). In the United States (U.S.), these behavioral risk factors and NCDs are more prevalent among low-income individuals and many racial and ethnic minority groups (Wang & Chen, 2011; Hawes et al., 2019; Schiller et al., 2012). Due to multiple socio-ecological barriers, evidence-based interventions to improve diet and physical activity patterns have had limited impact for these higher-risk populations, prompting calls for tailored interventions (Story et al., 2008; CDC, 2020; Bull et al., 2018; Morales et al., 2014; Ritchie et al., 2018; Kumanyika, 2018, 2019).

Constrained choice theory is a useful frame to understand the complex socio-ecological systems which contribute to these disparities (Bird & Rieker, 2008). This theory posits that decisions made at the government, community, work, and family levels shape health opportunities throughout the life course, resulting in choices that are often constrained and gendered (Bird & Rieker, 2008). For example, among racial and ethnic minorities, structural and income inequalities increase risk for unhealthy eating and activity patterns, and low-income groups experience barriers such as financial instability, unstable housing, and food insecurity (Hawes et al., 2019; Story et al., 2008; Satia, 2009). The impact of these constraints is even more profound for low-income, minority mothers, who often experience additional burdens such as extensive caregiving duties (Bianchi et al., 2000; McLoyd et al., 2000). This has lasting intergenerational effects, as mothers’ unhealthy diet and activity patterns further increase their children’s risk for these behaviors (Savage et al., 2007; Klohe-Lehman et al., 2007).

Tailoring diet and activity interventions for families with young children is therefore a high priority, and the first 1,000 days of a child’s life is an important window for establishing healthy diet and activity patterns (Blake-Lamb et al., 2016). A promising but underutilized strategy for interventions to reach and be tailored to low-income families, including families of color, is via home visitation programs (Salvy et al., 2017). Annually, over 500 publicly and privately funded models of home visitation programs (e.g., Healthy Families America, Early Head Start) provide services to more than 650,000 low-income families across the U.S. with diverse race and ethnic backgrounds. These programs are embedded in a comprehensive system of child and maternal health services and have successfully promoted optimal child development and improved maternal and infant health outcomes (Fergusson et al., 2006; Harding et al., 2007). However, HVs are not typically given training in evidence-based intervention components to promote healthy eating and physical activity (Salvy et al., 2017).

In home visitation programs, curriculum is provided in the home by a trained home visitor (HV), who frequently shares a similar culture and background with clients, and develops a close interpersonal relationship with them over a period of many months or years (Shanti, 2017). As Community Health Workers, HVs also work to support clients by directing them to outside community health systems, information, and resources (Scott et al., 2018; Witmer et al., 1995). Considering their connection to outside sources of information and their close relationships with clients, HVs have a unique, broad perspective of the diet and physical activity constraints that are faced by the low-income, racially and ethnically diverse families they serve (Witmer et al., 1995; Andrews et al., 2004).

There is a paucity of research documenting the challenges, strengths, and capacity building opportunities faced by families in home visitation programs in terms of adopting healthy eating and physical activity patterns. This study uses qualitative research to capture the rich and distinct perspective of HVs, to understand their experiences with the low-income, racially and ethnically diverse families whom they serve. Ultimately, the question is explored: “What are home visitors’ perspectives and experiences with their clients’ diet and physical activity behaviors?” The results can pinpoint key challenges and opportunities that could be targeted in future dietary and physical activity interventions within home visitation programs and other maternal and child health services.

## Methods

### Philosophical Assumptions

This study was based ontologically in relativism and in epistemological constructivism, with the perspective being that knowledge is constructed based upon participants’ understanding of their reality (Poucher et al., 2020). This study sought rich depictions of HVs’ experiences with their clients in the home visiting program, giving special attention to the role of society, culture, and power relations in shaping health.

### Parent Study

HV’s participating in this focus group study were part of an ongoing two-parallel arm randomized controlled trial, called “Healthy Habits” (R01HD092483–01; PI’s: de la Haye & Salvy), delivered through an established home visitation program (HVP) that served low-income mothers and their infants in California (Salvy et al., 2018; de la Haye et al., 2019). This HVP site offered services for mothers who were pregnant up until the child was five years old. Most clients are referred by a healthcare provider, the hospital or a community partner, or recruited at outreach events. All referrals are assessed and screened by the Family Resource Specialist or Supervisors for programmatic eligibility. HVs delivered curriculum in home, which focused on strengthening parent-child relationships and family functioning, promoting positive child development, and linkage to community resources (e.g., medical providers, financial/housing assistance, childcare, substance abuse treatment, community programs). Clients at this HV provider were usually low-income, racially and ethnically diverse (mostly Non-Hispanic White or Hispanic, with approximately 70% Hispanic), and represented a variety of household structures (e.g., some were single, and lived alone or with family; others lived with a partner, which were predominantly heterosexual).

The parent study, Healthy Habits, was used to recruit HVs for the focus groups, however it is important to note that the focus of the current study is not to evaluate the intervention. Rather, the goal is to explore these HV’s rich understanding of the diet and physical activity behaviors of all their clients, regardless of whether or not their clients were enrolled in the Healthy Habits program. The Healthy Habits curriculum was integrated into the existing home visiting program to provide nutrition and activity information, and focused on sustained adoption of healthy behaviors, and healthy weight trajectories, among mothers and their infants. HVs at this site were randomly assigned to deliver either the standard home visitation curriculum alone (control group), or the home visitation plus Healthy Habits curriculum (intervention group) for this trial. An initial training was conducted, where all HVs were given a broad overview of the Healthy Habits project including overall research process, timeline of the participant enrollment and study progression, how to introduce the study, how to complete the intake survey, and potential implications of the program. HVs in the control arm had no additional training related to diet and physical activity. HVs in the intervention arm were given an additional three-hour training on the implementation of Healthy Habits curriculum. Refresher training on the curriculum was offered about every 6 months. Mother and infant enrollment into the study was ongoing for one year prior, and HVs assigned to the intervention arm had been delivering the curriculum since that time, although they all had a mix of clients who were, and were not, enrolled in the parent study.

### Participants and Recruitment

Participants in this qualitative research are the HVs at the site of the parent study, who were among the first in the U.S. to receive training in nutrition and physical activity curriculum. All HVs in the intervention and control arms were invited to participate in a focus group via email from the project manager. Involvement was voluntary, and informed consent was obtained from all participants. Focus group attendees were given $10 gift cards as compensation for their time. Participating HVs (n=13) were all female and predominantly Hispanic or Non-Hispanic White. HVs had been working at the HVP community site for various durations, ranging from six months to 5+ years, with an average case load of 10–13 clients.

Fifteen HVs were participating in the Healthy Habits intervention/control groups and 13 agreed to participate in the first two focus groups. After reviewing the transcripts from these two groups, and identifying similar themes across both groups, it was determined that theoretical saturation had been reached and data collection was sufficient. Specifically, no new information was likely to be identified through further focus groups with the remaining HVs. All procedures were approved by the Institutional Review Board of University of University of Southern California (HS-18–00025).

### Procedure

To prevent cross-contamination of the ongoing intervention and explore differences in perspectives between groups, two focus group sessions were conducted in person: one session with control HVs (n=6); and one session with HVs delivering the Healthy Habits curriculum (n=7). Focus groups were chosen over other qualitative methods to allow for HVs to interact and build upon each other’s thoughts and stories. Both focus group sessions were conducted by the same interviewers in January of 2020, approximately thirteen months after the first participant to receive the Healthy Habits intervention had been enrolled. Due to the rolling enrollment of Healthy Habits participants, clients were all at different stages of the intervention, but none had fully completed the eighteen-month long intervention. Of the 44 participants who were enrolled in Healthy Habits, 26 were in the intervention, and 16 of these participants had been receiving the intervention for 9 months or less. Both focus groups lasted approximately 80 minutes and were conducted by the project manager and a graduate research assistant. Both interviewers had previously built rapport with the HVs through personal and professional interactions at monthly team meetings. The Healthy Habits project directors worked with the HVP site for over nine years and had built a trusting relationship with HVP staff but did not participate to reduce desirability bias. Thus, the focus groups took place in a relaxed and interactive context and provided a safe space for HVs to share their perspectives.

### Interview Guide

The interview guide was semi-structured, and questions were purposefully open and broad in scope, so that HVs were free to describe experiences across many socio-ecological layers and were not biased by the topics of the questions (Sparkes & Smith, 2013). The guide aimed to explore HVs’ perspectives and experiences with their clients’ constraints and capacity building opportunities for healthy diet and physical activity (Online Resources –supplemental materials). Questions were divided into four sections: (a) Introduction/General Health (e.g., “What were some of their most pressing concerns or questions about their health or their families’ health?”); (b) Physical Activity (e.g. “Can you tell me about times when moms were more interested in being physically active?”); (c) Healthy Eating (e.g. “Can you tell me about a time when a mom struggled with eating healthy?”); and (d) Closing Questions (e.g. “What would you do to support the health of your moms and their kids?”).

## Data Analysis

Focus groups were audio-recorded and transcribed verbatim. The focus groups were analyzed using the six steps of reflexive thematic analysis (Braun & Clarke, 2006):

1. The transcripts and audio-recordings were reviewed by the first author for accuracy, and to garner data familiarization.
2. Using NVivo qualitative analysis software (NVivo, n.d.), codes were identified inductively from the transcripts to identify meaningful units of data.
3. The final set of codes were reviewed to identify patterns of meaning, and organized into potential themes This examination of codes occurred within each transcript and across the control and intervention group transcripts to explore for similarities and differences. The same potential themes were identified in both groups.
4. The potential themes and coded extracts were reviewed, and the strongest and most well-supported themes were identified.
5. The final themes were reviewed, named, and refined.
6. The themes were organized into results and related back to the research question and relevant literature. To protect anonymity, pseudonyms were used in the reporting of results.

### Quality of Qualitative Analysis

Four quality indicators were chosen in light of the philosophical assumption of relativism (e.g. multiple context-dependent realities exist, and knowledge is constructed based upon one’s understanding of their reality), the theoretical model of Constrained Choice Theory, and the research question: (a) worthiness of the topic, (b) rich rigor, (c) credibility, and (d) significant contribution (Tracy, 2010). The worthiness of this topic was supported by the unprecedented rates of inadequate diet and activity, and the public health community’s recent focus on understanding the heightened risk of underserved families. Rich rigor was achieved through appropriate methods of data collection and collaborative and reflective analytic methods. Credibility was established in the current study through methods such as thick description (e.g. detailed description and interpretation), and through the selection of study participants – HVs who were reliable as key informants given their close relationships with their clients. Significant contribution included being a valuable addition to the scientific literature and the implications these findings have on diet and activity initiatives within home visitation programs.

## Results

The HVs in both the intervention and control group described similar themes. Based on the analysis of the focus groups, three overarching themes were identified: (a) acute periods of crises and mental health issues; (b) the role of the mother within the family; and (c) support and barriers within mothers’ broader social network. In addition to these themes, HVs emphasized that while their clients shared many common experiences, constraints, and strengths, they also differ in many ways and have unique needs. For example, HVs noted that some mothers experienced specific barriers, such as living in areas with decreased access to healthy food outlets and safe space for physical activity. As such, HVs voiced that behavioral interventions should be delivered one-on-one, which allows for programs to be tailored to each individual.

### Acute Periods of Crises and Mental Health Issues

The HVs discussed how mental health issues, such as depressive disorder, were common for their clients. Daily stressors such as a lack of transportation and constrained living situations, among others, contributed to their clients’ depression. Periods of crises and depression were closely intertwined, as symptoms of depression were often triggered or exacerbated by periods of acute crises. One example of an event that could trigger both an emotional and financial crisis for mothers was the death of a close family member. Angelina discussed a client who struggled emotionally with the grief and stress of losing her mother:

Her mom passed away, had cancer... Six months later she had the baby, but she felt like she couldn’t do anything because she had a five year old to take care of, a husband to take care of and, you know, trying to make ends meet... It was just trying to, you know, get stuff going, find a house, you know... It was like really a crisis, you know, for that family. (Angelina, 2020)

In this example, Angelina described how her client was living in her mothers’ home and depended on the shared resources, and how this magnified the emotional burden that her client experienced when her mother passed away. As Angelina expressed, the number and intensity of these difficulties left her client feeling overwhelmed and hampered her ability to take care of her and her family’s psychosocial and physical well-being.

HVs discussed how hard it was for their clients to invest in their health during periods of crises or depression. Some had initial successes with eating healthier and being more active, but when a crisis occurred, they no longer had the mental capacity due to more urgent priorities:

She was like really incorporating the healthy eating and the vegetables, and then, uh, they went through or they’ve gone through a period of financial stress where it’s like even just being able to make rent. It was a lot and she can’t work and dad wasn’t, you know, able to find full-time work... But when I would follow up with like how their healthy eating was going, she’s like, “I’m just gonna be honest. Like I just haven’t been able to keep up with it. I’ve been so stressed out. (Mariana, 2020)

HVs discussed how these experiences affected not only the health of their clients, but the health of their children as well. When undergoing depressive episodes, clients struggled to engage in active play with their children or cook healthy family meals (e.g. “Like they don’t want to cook ‘cause they don’t even want to get out of bed... It’d be easier to just give the kids fast food.”).

Another crisis that negatively impacted mothers’ mental health, as well as their children’s nutrition, was issues with federal assistance programs. Home visitors discussed their clients’ reliance on these programs, such as the Special Supplemental Nutrition Program for Women, Infants, and Children (WIC), a nutrition program serving low-income families with children under the age of five. One home visitor, Annie, gave the example of a client who struggled with having appropriate food options for her child due to issues with WIC:

When I visited her she had scooped the last of the formula out of the can and um, she said, “I don’t know when we’re going to be able to get more”... so when I came back the next week... She goes, “Yeah, we just haven’t had formula so we’ve been giving him table food”... And like the formula is very much like the sustaining part for him and so she was just like, “There’s nothing you can do and now I’m giving him table food that now I can’t give to my older kids.” She had to choose between her kids basically, like who’s gonna get this food because that’s how limited they are. (Annie, 2020)

As Annie and other home visitors noted, the loss of these services often resulted in a struggle for clients to provide adequate nutrition to their children. These experiences weighed heavily on HVs, and they felt that it was difficult to end visits with clients who were amid such intense crises. They also expressed guilt over addressing healthy eating and exercising with clients who were struggling with crises or depression:

I get the feeling like if I’m asking, asking someone to do something while they’re sick... So you kind of have to like feel out your mom and see who’s ready to have that, because it’s almost putting something else on their plate. (Mia, 2020)

HVs identified crises and depressive episodes as substantial barriers for clients and their families to engage in healthy eating and activity. However, as Mariana expressed, HVs believed that these barriers could potentially be overcome with adequate mental health services:

I recently saw her and she got back on medication after having the baby, and I can already see a difference in her energy and her motivation. She just seems, you know, happier. So I think that’ll, I’ll see a change there hopefully with her nutrition. (Mariana, 2020)

As such, HVs encouraged their clients to pursue mental health services and emphasized the emotional and physical benefits of receiving care.

### The Mother’s Role in the Family

The HVs frequently discussed the importance of family in the clients’ lives, and the role of the mother within her family. For most of their clients, traditional gender roles were adopted, with the mother being the primary caregiver for the entire family. They emphasized that for these mothers, the main priority was the needs and well-being of their children and partner, and that self-care was not always valued. This was especially true for Hispanic mothers. Here, Mariana talked about struggling to get clients to see the importance of investing time into their health:

Self-care is not something that is promoted within our culture. Especially in Hispanic cultures. It’s like once you become a mother, your focus is your children and your husband, if you happen to be married, and you’re kind of last... So kind of trying to get them into the mentality of it’s okay to take that time for yourself. It’s okay to take 30 minutes to either go for a walk, or it’s okay to eat a little bit healthier, prepare meals that are maybe not favorable to the children. (Mariana, 2020)

HVs also noted that some families maintained the traditional gender role of man as head of household. Sometimes, this created difficulties for mothers trying to implement healthy changes:

[In] Hispanic culture and things, it’s like mostly like what the man says goes... And so I feel like... if he was the one who made the rules a lot, it’s gonna be hard for her to be like, “No.” Like, “I’m gonna make the rules for dinner. (Andrea, 2020)

A few HVs expressed the belief that, even for their clients who practiced these traditional gender roles, it was still possible for them to cook healthier meals. They suggested that mothers cook multiple options, offering healthier alternatives for themselves and their children, and eating only small portions of their partner’s preferred dishes. Other HVs adamantly disagreed with this idea and insisted that it was not realistic for their clients to cook multiple family meals because mothers have to juggle several caregiving demands. Andrea described a typical dinner time scene that would make it too difficult for mothers to add this extra work:

It’s like they’re making the food and then they have to serve the, the husband and they have to heat up his tortillas and then they have to serve the kids... The kids are making a mess, and then they have to wash the dishes... ‘Cause that’s how the role is, that’s what the mom does during that time. (Andrea, 2020)

In light of this, HVs generally upheld the belief that feasible changes for their clients must be simple and noted that they had seen mothers be most successful with small changes such as practicing portion control or adding in movement throughout the day.

In line with clients’ roles as primary caregivers and the de-emphasis of self-care, HVs felt that they saw no value in trying to improve their own health unless it was also beneficial to their children. Thus, they often leveraged the child’s health as a motivator for their clients. One such opportunity for motivating healthy dietary changes was when their clients were breastfeeding:

I try to use that to keep them motivated. Like “your baby’s drinking, um, everything you’re eating. ‘Cause that’s what he tastes, he’s tasting all that. So, so try to eat healthy. If you don’t want to do for yourself, you know, do it for your baby, ‘cause I know you love your baby”. (Camilla, 2020)

Home visitors emphasized that their clients were much more motivated by their children’s health than their own. This was highlighted by Camilla’s story of a client who initiated healthy changes for herself and her whole family after she realized her son was struggling with his weight:

She comes home late from work. So I will get to her home and she’ll have like Kentucky fried chicken. Now I go there and she’s actually cooking. I could smell that she’s preparing for her kids. So now that her kids are getting older, she’s noticed that her oldest son is a little overweight. So he has a hard time, I’m sure, doing activities at school. She works in the school, so she’s able to see that. So now with her other two kids, the younger ones, I don’t think she wants that. (Camilla, 2020)

Camilla touched on how her client’s self-sacrificial values influenced her dietary choices, and how motivation for behavior change was closely tied to the perceived benefits to their children. However, HVs expressed that their clients were more frequently concerned with their children being underweight than overweight, and it was difficult to get clients to understand the importance of preventing excess weight gain. As Camilla stated, her client only noticed that her son was overweight because it was hindering his activity levels. Recognizing this, HVs sometimes took a different approach with clients who were more likely to prioritize educational benefits for their children over weight-related benefits:

For a walk, like tell them, you know, the colors when you go for walks or grocery shopping. Like just take extra time to walk around in the store to teach new stuff to the kids. So I kind of try to put it like a teachable moment for the kids, but it’s also some being active for them. (Camilla, 2020)

Other HVs agreed and stated that they tried to integrate physical activity into child developmental and educational activities whenever possible.

### Support and Barriers within Mothers’ Broader Social Network

HVs emphasized that for clients who had tried to adopt a healthy lifestyle, having a supportive social network—meaning the web of family, friends, and other individuals with whom they have interpersonal connections with in their daily life—was a key factor. Having at least one social network member that helped alleviate caregiving burdens enabled mothers to have more time and resources to pursue healthier lifestyles. A lack of this instrumental support was identified as a barrier, even for those who had previously engaged in healthy activities. As stated by Angelina, “They were pretty good, pretty active. But once they had the child, they have no one to babysit... they can’t take their babies to the gym.”

For these clients who were single and lived alone, their living situation created difficulties with balancing childcare responsibilities. For other mothers who lived in a multigenerational household, they received more help with household responsibilities and experienced less time constraints and less stress. HVs indicated that some of these mothers took advantage of this extra support by preparing healthy foods and exercising. However, living with family members sometimes decreased support, depending upon the structure of caregiving roles. When discussing whether having extended family in the household was helpful, Andrea said, “If they have like mom, like you know, like grandma, grandpa or someone there to help them, then yeah. But if the mom is like the caregiver for them also... has to be the sole supporter, then no.”

In addition to mothers’ needs for instrumental support and assistance with household tasks, HVs also highlighted their need for emotional and moral support. Unfortunately, they felt many clients received little emotional support. One common area in which mothers felt unsupported was in trying to make healthier family meals, as described by Andrea:

She said that she was trying to eat healthier, but it was hard because her younger son had autism. So then like he was, he’s really strict on like, you know, he has like one favorite thing he likes to eat, and her parents, since they were older, Hispanic, they didn’t want to eat like anything healthy... So for her it was really hard for her to incorporate things when like they’re both like so set on like what they want to eat and then, you know, she didn’t have any support so then she just gave up. (Andrea, 2020)

According to the HVs, conflict over family meals was even more deterrent when a male romantic partner was involved and almost always resulted in the mothers defaulting to his preferences.

The mothers’ social networks not only impacted their health through mechanisms of support, but also through social influence. Alexa provided an example of this and described how one mother struggled with unhealthy dietary behaviors when prompted by her partner:

She’s like, “You know, I try to be healthy but then we’re coming out of church and my husband sees the taco truck and he’s like, ‘Let’s go get some tacos’”. And it’s like 9:00 at night, she’s already had dinner but it’s like her fourth meal. (Alexa, 2020)

Due to the potential for social influence, HVs indicated the importance of getting the whole family involved in lifestyle changes, and discussed how it was especially beneficial when a romantic partner was pursuing healthy living too. In addition to these key family members, they indicated that mothers benefitted from being connected to peers outside of the family who were sources of positive influence.

## Discussion

### Acute Periods of Crises and Mental Health

A constraint that was discussed by HVs was acute periods of crises, and mental health issues, which were heavily intertwined. Among mothers living in poverty, depressive disorders are common, likely due to heightened daily stressors such as financial instability and lack of social support (Ammerman et al., 2010; Abrams & Curran, 2009; Chaudron et al., 2005). Though the prevalence of depression among low-income groups is well documented, the conceptualizations offered by the HVs give further insight into the multifaceted causes of this disparity. The HVs highlighted how precarious and fragile these families’ finances are and how one singular event that further constrains their finances can push them into a crisis. As the HVs discussed, these events varied among clients, and included things such as the death of a household member and the loss of federal services.

In parallel with the link between limited socio-economic resources and limited health opportunities outlined in Constrained Choice Theory, the HVs discussed how their clients’ financial barriers constrained their diet and activity (Bird & Rieker, 2008). These barriers directly constrained clients by leading to food insecurity and decreased access to healthy foods, and indirectly through depression (Blaine, 2008; Gillen et al., 2012). HVs discussed how difficult it was for mothers to overcome bouts of depression to cook healthy meals or exercise. Some mothers had initial successes, but when a crisis occurred, they no longer had the resources or the mental capacity, and other priorities felt more urgent. This also affected the health of clients’ children, echoing previous studies that have linked mothers’ depressive symptoms to adverse children’s health outcomes (Raposa et al., 2014; Thompson et al., 2018).

Though the HVs felt that periods of crises and depressive episodes were substantial barriers for these families’ health, they also expressed the potential for mental health services to lead to multifaceted improvements. HVs stated that mothers who were receiving reliable mental health support and services were better prepared to improve their family’s health behaviors.

### The Mother’s Role in the Family

The discussions in this study highlight how various socio-ecological influences may create gendered constraints that limit healthful opportunities (Bird & Rieker, 2008). The mothers being described in this study were often at the center of the family, which created difficulties for implementing healthy changes. This is in line with previous literature documenting that primary caregivers for young children tend to feel overwhelmed with demands and time constraints and that this often leads to decreased healthiness of family meals (Bianchi et al., 2000; McLoyd et al, 2000; Mills et al., 2017). Though it’s very common for mothers of all cultures to prioritize housework and child care duties above self-care, the “self-sacrificial” mindset discussed in this study is more prominent among Hispanic mothers due to the cultural value of “marianismo” ((Bianchi et al., 2000; McLoyd et al, 2000). “Marianismo” celebrates a mother’s role as caregiver and emphasizes selflessness (McLoyd et al., 2000; D’Alonzo, 2012). For women who have been taught this cultural value, self-care can be viewed as a selfish indulgence because investing time in one’s self results in lost time they could be allocating to their family’s needs (D’Alonzo, 2012).

As such, HVs suggested harnessing clients’ children as motivation for healthy changes. This perspective is consistent with prior work documenting how mothers’ motivation for behavior change is closely tied to the perceived benefits to their children (MacMillan Uribe & Olson, 2019). However, many low-income mothers are not concerned with their child being overweight, but rather underweight, and in Hispanic culture, heavier babies are often seen as healthier babies (Jain et al., 2001; Martinez et al., 2017). These beliefs can lead to overfeeding and shift the focus away from feeding one’s children nutritious foods (Jain et al., 2001; Martinez et al., 2017). Mothers who hold these beliefs may not change their feeding practices until excess weight has reached such a level that daily activities are hindered, and one HV discussed a similar scenario with one of her clients (Jain et al., 2001). The HVs discussed alternative strategies for harnessing their clients’ children as motivators, such as integrating physical activity into educational activities.

### Support and Barriers within Mothers’ Broader Social Network

The findings of this study reflect extensive literature documenting the importance of one’s social network in relation to eating and physical activity (Zhang et al., 2018; Strine et al., 2008). Uniquely, the HVs in this study discuss how having just one social network member who was a positive connection was beneficial. These positive connections came in many forms, from a network member who babysat so mothers could cook or exercise, to someone who co-engaged in exercise.

Low levels of support have been linked with obesity, and support is critical when one is seeking to improve dietary quality or physical activity levels (Zhang et al., 2018; Strine et al., 2008). The HVs identified help with childcare and household duties as a critical area in which their clients needed instrumental support. For mothers without this, the constraint of juggling extensive caregiving duties prevented them from engaging in positive health behaviors, as has been documented in prior studies (Bianchi et al., 2000; McLoyd et al, 2000). Another important barrier identified was disagreement over family meals, a common barrier for mothers who often seek to avoid this type of “unnecessary” conflict and feel a sense of connection when sharing family meals that everyone appreciates and enjoys (Homish & Leonard, 2008; Bove et al., 2003).

Male romantic partners were identified as a crucial member of mothers’ social networks. According to the HVs, conflict over family meals was even more deterrent when a male romantic partner was involved and almost always resulted in the mothers defaulting to his preferences. HVs also expressed that partners were negative influences on some of their clients, by prompting them to eat unhealthy foods. This is in line with previous literature indicating the potential for partners to serve as barriers for lifestyle changes (de la Haye et al., 2019; Zhang et al., 2018). Additionally, this sheds light on previous work with this population which indicated that cohabitating with a spouse or partner was associated with worse maternal intervention outcomes (de la Haye et al., 2019).

Taken together, these findings reinforce previous literature that emphasizes the importance of one’s social network in terms of changing dietary patterns and physical activity. Thus, interventions should foster new positive connections, while also leveraging and targeting participants’ important others to promote healthy changes.

### Limitations

The results of this study are based off a small sample size, limiting the generalizability to HV clients at other locations. If efforts to address diet and activity gaps in home visitation services are systematically adopted at other sites, more research would be needed to understand the perspectives and experiences of HVs at these locations. As the focus groups were conducted during the early stages of the intervention, this study could not assess the effectiveness of the intervention, and there were no differences in the themes identified by the HVs in the intervention arm and the HVs in the control arm. It would be valuable for our intervention study, and others, to gather data at the completion of an intervention, to explore differences across study arms and identify new challenges and opportunities that may arise.

### Implications

The unique perspective of HVs and the themes identified in the current study highlight the complexity of health needs and opportunities of the diverse clients enrolled in home visitation programs. In line with Constrained Choice Theory, these themes emphasized that these mothers’ diet and activity decisions do not exist in isolation but are interacting with multidimensional influences, including socioeconomic and social constraints. These barriers distinctly affected mothers, who struggled with the added stress and pressure of being primary caregivers.

Despite these constraints, HVs were optimistic and hopeful that their clients could make improvements if provided with adequate support and information, including through health interventions. As it is not standard for HVs to be trained to provide diet and activity curriculum, future interventions within HV programs would likely require additional training, similar to the training offered in the Healthy Habits program. When discussing opportunities for health interventions, HVs favored simple and one-on-one interventions that targeted the entire family and could be tailored to families’ diverse needs and cultures. The Healthy Habits program aligns with these preferences, but both the intervention and control HVs emphasized some other common social and financial barriers that are not addressed by the Healthy Habits program. For example, Healthy Habits targets maternal social networks through communal classes, where mothers in the program can connect with one another and form new social ties. However, HVs also highlighted the importance of involving other key network members, such as romantic partners. Future interventions within home visitation programs should consider including mothers’ important network members, such as partners or household members, and consider the other social and financial barriers outlined in this study.

Broader community and policy level initiatives may also facilitate the success of future nutrition and activity interventions. Mental health barriers were repeatedly highlighted by HVs, and though HVPs work to connect clients to mental health care providers, a lack of transportation or long wait times for appointments often hindered care. Community level initiatives to improve public transportation, or to increase the number of local mental health care providers, could remove some of these barriers to care. At the policy level, expanding and increasing food assistance programs may better enable home visitation clients to improve their diet, as HVs emphasized the vitality of programs such as the Supplemental Nutrition Assistance Program (SNAP) and WIC. These expansions could include increasing monthly allowances, allowing noncitizens to access services, and offering greater flexibility with scheduling appointments. HVs stated another common barrier was a lack of affordable and safe places for physical activity; thus, policies that improve access to park and recreational facilities may also enable these families to engage in more regular activity. Taken together, the findings outlined in this study suggest that future initiatives with clients enrolled in HVPs will require multilevel initiatives that address the individual, social, community, and policy level barriers to healthy lifestyles.

## Supplementary Material

Supplemental Materials - Interview Guide
