# Supplementary material for: Diet and Physical Activity Behaviors of Families Receiving Maternal and Child Health Services: The Perspective of the Home Visitor
Source: J Healthy Eat Act Living. Author manuscript; Available in PMC 2022 Aug 3. (PMC9348138; doi:10.51250/jheal.v2i1.33)
Supplement: Supplemental Materials - Interview Guide [file NIHMS1808513-supplement-Supplemental_Materials_-_Interview_Guide.docx]

**Appendix I – Focus Group Interview Guide**

**Opening Questions – General Health**

*To start with, I have a couple questions about how your moms think about health and wellbeing.*

Think back to some of the times you’ve discussed health with the moms you are working with. How do you think they defined “being healthy” for themselves? What about for their children?

- What were some of their most pressing concerns or questions about their health or their families’ health?

Compared to other priorities in their life how important do you think health is to your moms?

**Physical Activity**

*Now, I’d like us to talk about physical activity and what it’s like for the moms you are working with. Physical activity can be any movement that moms do throughout the day, like gardening, exercising, walking, dancing, etc.*

- Think back to times you’ve discussed physical activity with the moms you are working with. How do you think they defined “physical activity” for themselves? What about for their children?
- Compared to other priorities in their life how important do you think being physically active is to your moms?
- Can you tell me about times when moms were more interested in being physically active?
- What would your moms need to be more active?
- Are your moms seeking or interested in opportunities to be physically active with their children or family members?

Facilitator NOTE: Next set of questions for intervention group only. *Now, I’ll ask you a few questions that are about the Healthy Habits material on physical activity…*

- Thinking back when you introduced physical activity as part of Healthy Habits, what was most useful for moms?
- And was there anything in the physical activity material in Healthy Habits that was less useful or difficult to understand?
  - Prompt (if needed): For example, was there a time when your moms had a hard time changing her physical activity habits?

**Healthy Eating**

*Now, I’d like us to talk about eating healthy - what it means and what experiences your moms are having. First I am going to focus on the mom’s experience then we will talk about the children.*

- First, what does “eating healthy” mean to the moms you work with?
  - Prompt if necessary –
    - Can you give me an example?
    - Where do you think these beliefs come from?
- Since you began working with your moms how has their thinking about “eating healthy” changed?
- How would your moms describe “eating healthy” for their babies and infant children?
  - Prompt if necessary –
    - Where do you think these beliefs come from?
- Can you tell me about a time when a mom struggled with eating healthy?
- Can you tell me about a time she struggled with feeding her children in a healthy way?
  - Probe: Why did she seem to be struggling?
- What do you think your moms need in order to eat healthy?
- Can any of you tell me about a time when a mom was more interested in eating healthy?
- Can any of you tell me about a time when a mom was focused on feeding her children in a healthy way? In other words, a time when this was especially important to her?
  - Why do you think she felt this way at that time?
    - Prompt: What was happening at the time?

Facilitator NOTE: Next set of questions for intervention group only. *Now, I’ll ask you a few questions that are about the Healthy Habits material on healthy eating…*

- Think back to when you introduced the Healthy Habits material on decreasing sugary beverages and fried foods, and increasing vegetables and fruits. What parts of this material was most useful for moms?
  - Prompt: Can you give me some examples?
  - Prompt (if not mentioned): Are there examples of how this was helpful for mom’s eating habits, or their child’s eating habits?
- And were there parts of this material, on sugary beverages, friend foods, and fruits and vegetables, that was less useful or difficult to understand?
  - - Prompt: Can you give me some examples, like any time when the mom had a hard time changing her eating habits, or their child’s eating habits?
- Overall, have other family members become involved in learning about or doing any of the Healthy Habits materials you give the moms?
  - Prompt: Can you give me some examples?

**Closing Questions**

*Now, we want to focus a little more on you and the experiences you have had.*

- (Intervention group only) In what ways have you been influenced by the Healthy Habits program, since you started delivering it?
  - *Prompt for those who say they have been influenced:* So ___, you said that you have gotten a lot out of the program. Does this seem to have rubbed off on any of your moms, and if so, how?
- (Intervention group only) If Healthy Habits was your program, what are the things that you would do differently?
- Now let’s have fun with this last question… imagine that you are Mayor for today and any decisions you make today will last forever. There are no limits on what you can do!:
  - What would you do to support the health of your moms and their kids?
    - Prompts: Eating Healthy, Physical Activity
- Before we end for today, is there anything that we have missed that you think we should know?
